# Dopamine D1 receptor density in the mPFC responds to cognitive demands and receptor turnover contributes to general cognitive ability in mice

**DOI:** 10.1038/s41598-018-22668-0

**Published:** 2018-03-14

**Authors:** Christopher Wass, Bruno Sauce, Alessandro Pizzo, Louis D. Matzel

**Affiliations:** Department of Psychology, Program in Behavioral and Systems Neuroscience, Rutgers University, Piscataway, NJ 08854 USA

## Abstract

In both humans and mice, performance on tests of intelligence or general cognitive ability (GCA) is related to dopamine D1 receptor-mediated activity in the prelimbic cortex, and levels of DRD1 mRNA predict the GCA of mice. Here we assessed the turnover rate of D1 receptors as well as the expression level of the D1 chaperone protein (DRiP78) in the medial PPC (mPFC) of mice to determine whether rate of receptor turnover was associated with variations in the GCA of genetically heterogeneous mice. Following assessment of GCA (aggregate performance on four diverse learning tests) mice were administered an irreversible dopamine receptor antagonist (EEDQ), after which the density of new D1 receptors were quantified. GCA was positively correlated with both the rate of D1 receptor recovery and levels of DRiP78. Additionally, the density of D1 receptors was observed to increase within 60 min (or less) in response to intense demands on working memory, suggesting that a pool of immature receptors was available to accommodate high cognitive loads. These results provide evidence that innate general cognitive abilities are related to D1 receptor turnover rates in the prefrontal cortex, and that an intracellular pool of immature D1 receptors are available to accommodate cognitive demands.

## Introduction

Among humans, 40–50% of the variance of individuals’ performance across diverse cognitive tasks can be accounted for by a single “general” influence^1,2^. A similar general cognitive factor accounts for 30–40% of the variance in the performance of individual mice across batteries of 6–9 cognitive tasks^3–6^. Although variations in intelligence are likely to reflect multiple influences^7^, it is well established (in both mice and humans) that working memory capacity plays a central role in determining these variations^8–11^.

It has been reported that at least nine genes (of 25,000 screened) are up-regulated in the prefrontal cortex (PFC) of mice that express high general cognitive abilities (GCA), and three of those genes (Drd1a, Darpp-32, and Rgs9) comprise a cluster involved in dopaminergic signaling^12^. Relatedly, in humans the dorsolateral PFC (dlPFC) is engaged during working memory-based tasks and is differentially activated by cognitive demands in persons of high and low intelligence^13,14^. Furthermore, D1 receptors in the PFC are a target for working memory training in both humans^15–17^ and mice^18^. Lastly, mice with high GCA exhibit increased neuronal activation in the mPFC in response to D1 agonists^18^. In combination, these results suggest that general cognitive performance, working memory, and dopamine signaling in the PFC are related, and comprise a system that contributes to variations in intelligence^19–21^.

Although the Drd1a gene is upregulated and the responsivity of D1 receptors is elevated in animals that express high GCA, previous work has not detected a corresponding increase in membrane-bound D1 protein in the PFC^18^, suggesting that the number of mature D1 receptors does not contribute to variations in GCA. Rate of protein turnover, variations in chaperone proteins, and gene silencing are regulatory mechanisms that modulate the synthesis and trafficking of newly synthesized receptors to the plasma membrane^22^. Synthesis of the D1 receptor begins in the endoplasmic reticulum (ER). Once folded, the receptor is trafficked to the Golgi apparatus and then the trans-Golgi network by chaperone proteins, undergoes glycosylation, and can then be inserted in the plasma membrane. Upon ligand binding, the receptor undergoes degradation or is recycled back to the plasma membrane^23^. The balance of these cascades determines the level of receptor expression at the membrane. Since newly synthesized receptors are more responsive to transmitter binding, these regulatory mechanisms can influence the cell’s response to transmitter binding independent of receptor density^24^.

The dopamine receptor interacting protein, DRiP78, is a ubiquitous regulator of the export of immature receptors from the ER to the Golgi complex and their subsequent trafficking to the plasma membrane^25^, and elevated levels of DRiP78 promote sequestration of immature D1 receptors in the ER^25^. It is plausible that under cognitive resting conditions (when an increased number of D1 receptors are not needed), DRiP78 maintains an intracellular pool of immature receptors that await transport on an as-needed basis (e.g., in response to cognitive demands). Relatedly, variations in the rate of protein turnover could contribute to the efficacy of D1 signaling, as newly inserted receptors increase the cell’s response to dopamine^24^. It is plausible that animals expressing high GCAs have an increased rate of receptor turnover which would re-sensitize the cell at a faster rate, requiring an increased rate of receptor synthesis (accommodated by the increase in Drd1A mRNA).

Here (in Experiment 1) we test the possibility that under cognitive resting conditions, animals exhibiting high GCA express an increased rate of receptor turnover and elevated levels of DRiP78 (indicative of a larger intracellular pool of receptors awaiting recruitment to the plasma membrane). Rate of turnover of the D1 receptor was assessed after administration of *N*-ethoxylcarbonrl-2-ethoxy-1,2-dihydroquinoline (EEDQ). EEDQ induces a dose-dependent depletion (through irreversible non-competitive binding) of dopamine D1 and D2 receptors (with markedly lower affinity for other monoamine receptors)^26–28^. A specific antibody for D1 receptors was then used to monitor the rate of recovery of new D1 receptors. This strategy has been widely used to assess the regional and age-dependent differences in the recovery and turnover of DA receptors^28–30^. In addition (in Experiment 2), we assess whether high cognitive demands can promote a rapid increase in the density of mature D1 receptors. In both cases, analyses were performed on the medial prefrontal cortexl (mPFC), where prior work has found the highest correspondence between D1 receptor activity and general cognitive abilities.

## Results

### Experiment 1

Here we assessed the rate of D1 receptor turnover and levels of DRiP78 in the medial PFC of mice that had been characterized for GCA. Initially, the mice were characterized for their aggregate cognitive performance across a battery of four learning tasks. A principal components analysis was then applied to the animals’ performance on these four tasks in order to derive each individual animal’s factor score (a measure of each animal’s aggregate, or general, learning performance). Following characterization of all mice’s general cognitive abilities, they underwent a 14 day “rest” period to allow them to reach a state of cognitive “rest”. Subsequently, each mouse was administered the irreversible dopamine D1 receptor antagonist N-Ethoxylcarbonrl-2-ethozy-1,2-dihydroquinoline (EEDQ). Receptors present in the plasma membrane after the injection of EEDQ would be indicative of newly inserted receptors. After injection of EEDQ, animals were sacrificed at three time points (24, 72, and 288 hours), and the density of D1 receptors as well as DRiP78 levels were determined.

A Pearson’s product-moment correlation matrix was created from the mice’s rate of acquisition on all learning tests (Table 1), which revealed a positive manifold, i.e., all correlations were consistently positive (a result that suggests a common, i.e., general, source of variance). The Pearson’s correlation matrix was then subjected to a principal components analysis. All tasks loaded consistently and positively on a primary factor. This factor had an eigenvalue of 1.24, and accounted for 31% of the variance in performance across all tasks (Table 2), again suggesting a single general influence on the performance of mice on all learning tasks. Based on this primary factor, we derived a factor score for each individual mouse which served as a measure of each mouse’s general cognitive ability. Factor scores ranged from −1.65 to +2.7, where higher values reflect higher GCA. The factor scores were then used to construct three groups of animals that represent the top third, middle third, and bottom third of the distribution of general cognitive abilities. Mice from the middle third of the distribution would receive no injection of EEDQ, and thus could serve as a baseline against which to assess differences in D1 protein or DRiP78 levels.

**Table 1:** Correlation matrix for 98 animals from Experiment 1.

	LM	PA	WM	OD
Lashley III Maze (LM)	1.00	0.5	0.32	0.15
Passive Avoidance (PA)	0.5	1.00	0.21	0.08
Water Maze (WM)	0.32	0.21	1.00	0.12
Odor Discrimination (OD)	0.15	0.08	0.12	1.00

**Table 2:** Unrotated Principal components analysis for 98 animals from Experiment 1.

	General Cognitive Ability
Lashley III Maze	0.78
Passive Avoidance	0.63
Spatial Water Maze	0.45
Odor Discrimination	0.21
**eigenvalue**	1.24
**Variance Explained**	31%

**Figure 1:**
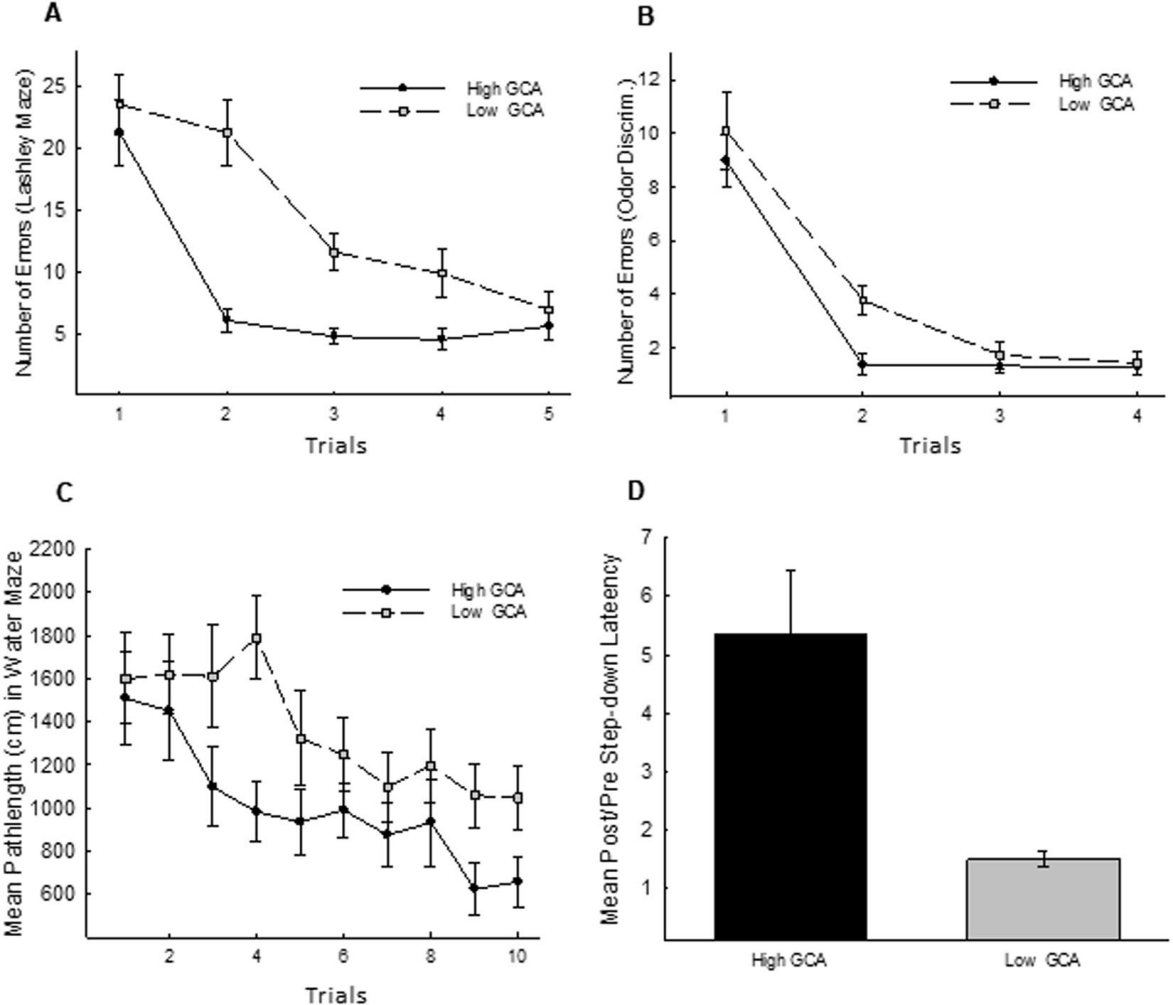
Cognitive performance of high and low GCA animals. Based on a principal component analysis of all learning data, 98 animals were classified according to their aggregate performance (general cognitive ability; GCA) across four learning tasks. The animals were then segregated into the top, middle, and bottom thirds (high, intermediate, or low) GCA. In all panels of Fig. 1, the mean performance of the high (n = 29) and low (n = 28) GCA animals is illustrated on each of the learning tasks. High GCA animals outperformed low GCA animals on each of the four tasks: Lashley III Maze [**Panel A**], odor-guided discrimination [**Panel B**], Morris Water Maze [**Panel C**], and passive avoidance [**Panel D**]). Brackets indicate standard error of the mean.

The performance of the high (n = 29 of a total of 98 animals tested) and low (n = 28) GCA animals on each of the learning tasks is illustrated in Fig. 1, and ANOVA was used to compare the performance of these two groups on each task. On all tasks, mice classified as expressing low GCA exhibited a slower rate of learning, with significant differences in the Lashley III maze, F(1,55) = 5.28, P < 0.001 (See Fig. 1A), the odor discrimination task, F(1,55) = 7.23, p < 0.01 (See Fig. 1B), the spatial water maze, F(1,55) = 9.22, p < 0.01 (See Fig. 1C), and passive avoidance, F(1,55) = 12.41, P < 0.001 (See Fig. 1D).

After completion of behavioral testing we assessed whether the rate of D1 receptor turnover and levels of the ER chaperone protein DRiP78 were related to animals’ general cognitive performance. Two weeks following the completion of the learning battery, the animals that were assessed for general cognitive abilities were subdivided into three groups of highest (n = 29), middle (n = 23), and lowest (n = 28) GCA. The 23 animals closest to the center of the distribution were used as a vehicle-treated control group in order to derive a baseline measurement of DRiP78 and D1 receptors. The highest and lowest GCA animals (ns = 29 and 28, respectively) were administered an injection of the irreversible dopamine antagonist EEDQ, and were sacrificed at one of three independent time points, 24, 72, and 288 hours (1, 3, and 12 days) after the injection. Since EEDQ is an irreversible receptor antagonist, D1 receptors detected at 24, 72, and 288 hours would be indicative of receptors synthesized after EEDQ injection. Thus, the number of receptors at these post-injection time points would provide an estimate of rate of receptor turnover/recovery. Levels of D1 receptors and DRiP78 were then quantified using enzyme linked immunosorbent assays. It is noted that the antibody used here to quantify D1 protein binds to the protein’s C-terminus, a binding site that is typically occupied by DRiP78 in sub-membrane immature receptors. Thus immature receptors awaiting transport to the membrane are undetectable by this antibody.

**Figure 2:**
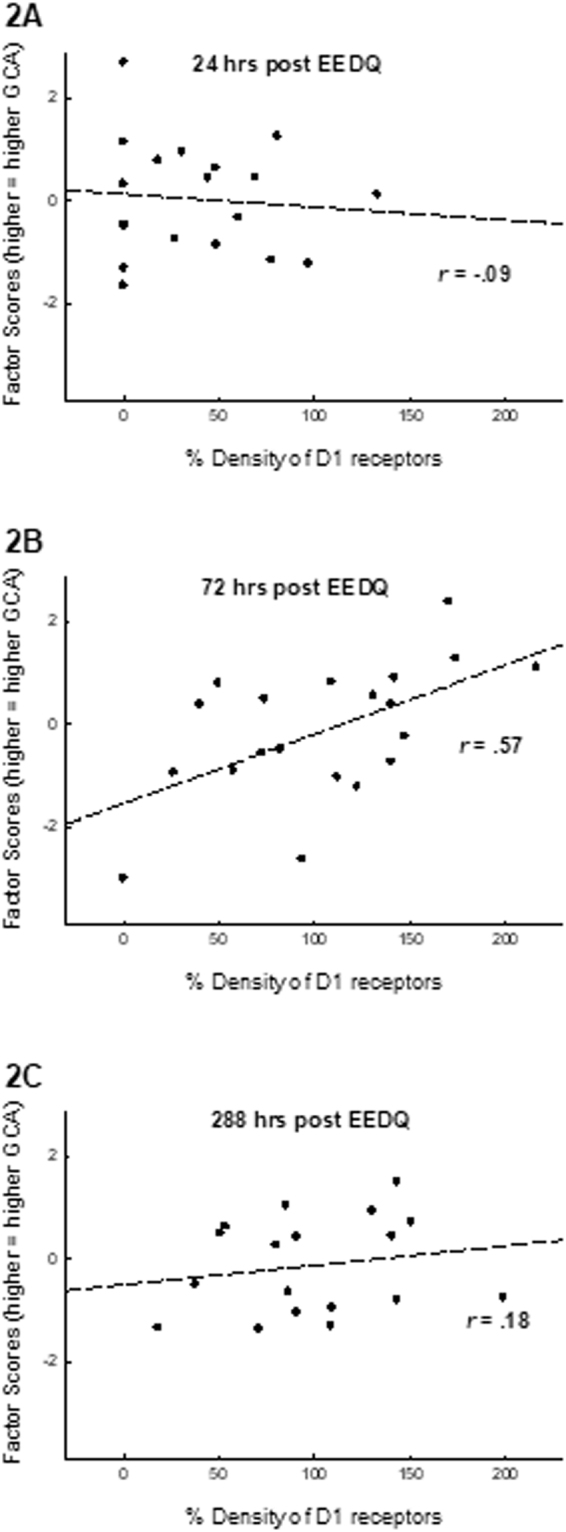
Recovery rate of D1 receptors as a function of GCA. Animals from the high and low GCA samples (see Fig. 1) were administered EEDQ (8 mg/kg), which irreversibly binds the D1 receptor. Receptors that can be detected after EEDQ injection are indicative of newly available receptors. Control animals received vehicle injections and served as a baseline from which to normalize protein levels. All animals were then sacrificed 24 hours, 72 hours, or 288 hours (12 days) after the injection of EEDQ. D1 receptors and DRiP78 in the mPFC were estimated using a DRD1 ELISA kit and DRiP78 ELISA kit. **Panel A**: 24 hours after EEDQ administration, D1 receptor levels were substantially reduced, and no relationship was observed between D1 levels and animal’s general cognitive performance. **Panel B**: Partial recovery of D1 receptors was observed 72 hrs after EEDQ, and the the degree of recovery was related to animal’s GCA, *r* (18) = 0.57, p = 0.009, such that high GCA animals expressed a higher density of new D1 receptors. **Panel C**: By 288 hrs after EEDQ administration (when recovery of D1 receptors would have been complete), no relationship was observed between D1 levels and GCA. In total, this analysis indicates that under normal resting conditions, D1 levels are not related to GCA, while the rate of turnover of the D1 protein is positively related to GCA.

Correlations between D1 levels and factor scores (indicative of GCA) were computed for all mice (those that received EEDQ injections) at each of three time points (24, 72, and 288 hrs) after the administration of EEDQ. The D1 levels were normalized relative to those mice that did not receive the EEDQ injection. Figure 2 presents the correlation matrixes obtained at each time point. Figure 2A indicates that D1 levels were significantly reduced 24 hrs after EEDQ (mean 38.6% ± 9.06% of baseline levels), and no correlation was observed between factor scores (GCA) and D1 levels, *r* (17) = −0.09, ns, 95% CI [−0.55, 0.41]. Similarly, no correlation between D1 levels and factor scores was observed 288 hrs after EEDQ, a point at which D1 receptors were fully recovered (mean 99.3% ± 10.8% of baseline levels), *r* (16) = −0.18, ns, 95% CI [−0.62, 0.35]. However, 72 hrs after EEDQ, a point at which D1 receptors were in the process of recovery, a significant relationship between D1 levels and factor scores was observed, *r* (18) = 0.57, p = 0.009, 95% CI [0.14, 0.82]. This latter result is indicative of faster recovery of the D1 receptor among mice expressing higher GCA. Furthermore, Fisher r-to-z transformations showed that the correlation of D1 levels and factor scores at 72 hrs was significantly higher than the correlations at 24 hrs, z = 1.99, p = 0.046, as well as at 288 hrs, z = 2.19, p = 0.028.

**Figure 3:**
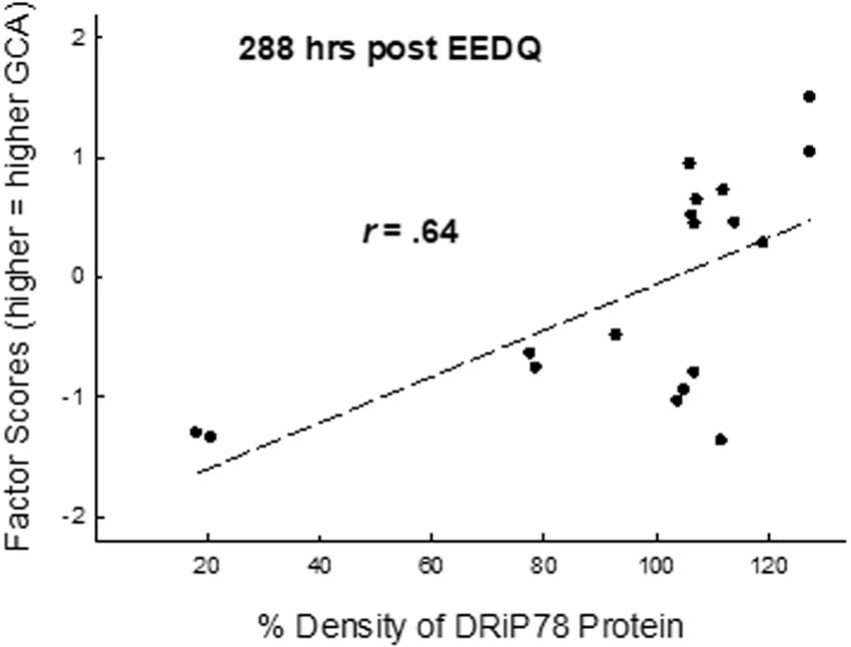
DRiP78 levels as a function of GCA. DRiP78 levels in the mPFC were estimated by ELISA. Samples were obtained from the same animals represented in Fig. 1 288 hrs after EEDQ administration (a time at which D1 levels had completely recovered). DRiP78 levels were positively correlated with animals’ GCA, *r*(18) = 0.64, p = 0.004, suggesting that higher levels of DRiP78 would support an increased number of sequestered immature D1 receptors that could be rapidly inserted into the membrane. It is noted that two points in Fig. 3 appear as outliers, i.e., two animals with very low GCA also expressed extremely low DRiP78 values. If these two animals were removed from the analysis, the correlation between DRiP78 levels and GCA remained intact, *r*(16) = 0.58, p = 0.018.

The results of the analysis of DRiP78 expression levels are illustrated in Fig. 3. DRiP78 levels were determined at 288 hrs, a point at which D1 protein had recovered from the EEDQ injection. As with the analysis of D1 levels, DRiP78 were normalized relative to those mice that did not receive the EEDQ injection. As can be seen in Fig. 3, DRiP78 levels were strongly related to animals’ factor scores (GCA), *r* (16) = 0.64, p = 0.004, 95% CI [0.21, 0.86]. It is noted that in Fig. 3, two samples appear as outliers, expressing only ~20% of the Drip78 level of controls. If these animals are removed from this analysis, the correlation between DRiP78 and factor scores remains high, *r* (14) 14 = 0.58, p = 0.018, 95% CI [0.07, 0.85]. This analysis indicates that DRiP78 levels are elevated in mice that express higher GCA, raising the possibility that a larger pool of sequestered D1 receptors is available for insertion into the membrane in response to cognitive demands.

### Experiment 2

In this experiment, mice underwent assessment in the learning battery described in Experiment 1, after which they were divided into two groups, matching the groups based on animals GCA. One group was then exposed to a brief period of intense working memory utilization^18,31^. The second group of mice was allowed an equal amount of time to explore the working memory training apparatus without any explicit working memory demands. Two hours after the completion of the working memory or control regimens, the animals were sacrificed and assessed for the density of mature D1 receptors levels in order to determine whether the imposition of a working memory demand elevated D1 protein levels, i.e., would a pool of intracellular D1 receptors (as implied by Experiment 1) be available to meet the demands of intense utilization of working memory.

Prior to working memory training, animals’ performance was once again measured during the acquisition phase of training on four learning tasks. In order to characterize each animal’s general cognitive abilities, a Pearson’s product-moment correlation matrix was created from the learning battery data (Table 3), which revealed a positive manifold, i.e., all correlations were consistently positive, suggesting a general influence across all learning tasks. The Pearson’s correlation matrix was then subjected to a principal components analysis. A primary factor (indicative of general cognitive performance) was extracted by the principal components analysis with an eigenvalue of 1.72, which accounted for 43% of the variance (Table 4). Based on this primary factor, we derived a factor score for each individual animal which served as a measure of each animal’s general cognitive ability. Factor scores ranged from −1.85 to +1.92, where higher values reflect higher GCA.

**Table 3:** Correlation matrix for 32 animals from Experiment II.

	LM	PA	WM	OD
Lashley III Maze (LM)	1.00	0.35	0.44	0.27
Passive Avoidance (PA)	0.35	1.00	0.07	0.24
Water Maze (WM)	0.44	0.7	1.00	0.03
Odor Discrimination (OD)	0.27	0.24	0.03	1.00

**Table 4:** Unrotated Principal components analysis for 32 animals from Experiment II.

	General Cognitive Ability
Lashley III Maze	0.85
Passive Avoidance	0.64
Spatial Water Maze	0.57
Odor Discrimination	0.50
**eigenvalue**	1.72
**Variance Explained**	43%

Once the animals’ factor scores were obtained the animals were divided into two groups (WMT and EXP; see below) that were matched based on factor scores, i.e., each group was constructed of animals of similar mean and range of general cognitive abilities (mean factor score of Group WMT = 0.07 ± 0.33; mean factor score of Group EXP = −0.08, ±0.23).

After the two groups were matched for GCA, one group of animals underwent a procedure intended to tax working memory (Group WMT). The second group of animals (Group EXP) was not required to utilize working memory, but instead was allowed to explore the working memory test apparatus for the average amount of time that each of the subjects from Group WMT was in the apparatus.

**Figure 4:**
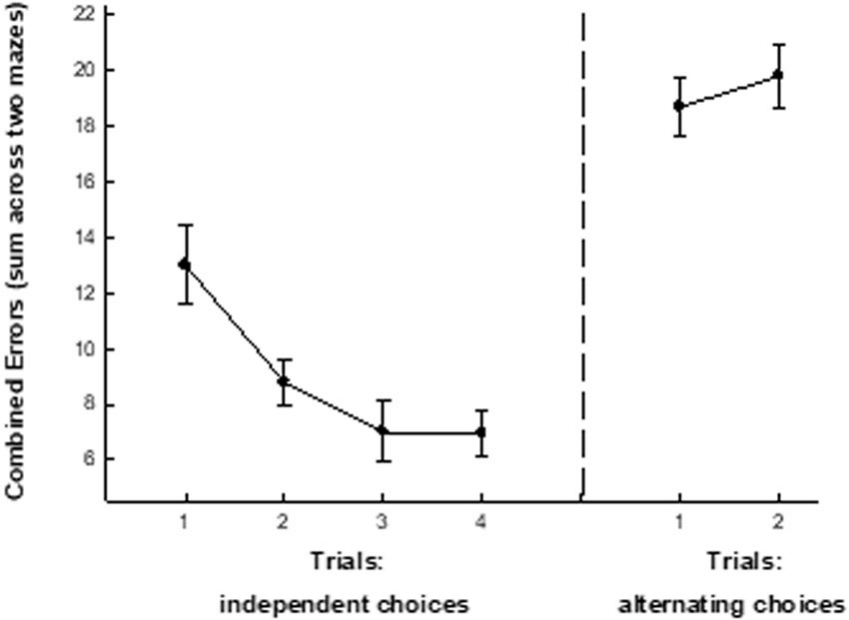
A Working memory test taxes the cognitive capacity of mice. Animals underwent a procedure that was designed to acutely tax working memory capacity. All animals were trained on two 8-arm radial mazes (one black, one grey). After reaching asymptotic performance in each maze, the mice were then required to perform in the two mazes in succession (performing in two mazes each day for four days). The **left panel of** Fig. 4 illustrates (mean ± SEM) the combined performance in the two mazes on each of four trials. When required to make these independent choices in the two mazes, animals’ performance was initially poor, then improved across the four days of training. Subsequently, animals were required to perform in both mazes, but alternated choices across the mazes (i.e., 3 choices in the black maze were followed by 3 choices in the grey maze, and this pattern repeated until all food was recovered). This “alternating maze” task has been asserted to tax working memory, as the animals must maintain a memory of the choices in each maze while making choices in the alternate maze. Since the guidance cues are common to both mazes, this procedure introduces a high level of interference and confusion. The **right panel of** Fig. 3 illustrates (mean ± sem) the performance of the animals during two alternating choice trials. Relative to the simpler task of making independent choices in two mazes (**left panel**), alternating choices in the two mazes promoted a severe decrement in performance (i.e., an increase in combined errors in the two mazes).

A dual-maze procedure was used to promote utilization of working memory. Figure 4 illustrates the performance of Group WMT during Phase 5 of training in which the animals performed successively in the black and grey mazes each day for four days (making only minimal demands on working memory). Performance during this phase of training (averaged across the two mazes, left panel) showed a steady improvement across the four days of training. However, when the animals were subsequently required to alternate choices across the two mazes (acutely taxing working memory), performance was severely degraded (averaged across the two mazes; right panel), and no improvement in performance was observed across the two testing days. Thus it can be assumed that animals were in a state of high cognitive demand at the completion of the second day of working memory implementation.

**Figure 5:**
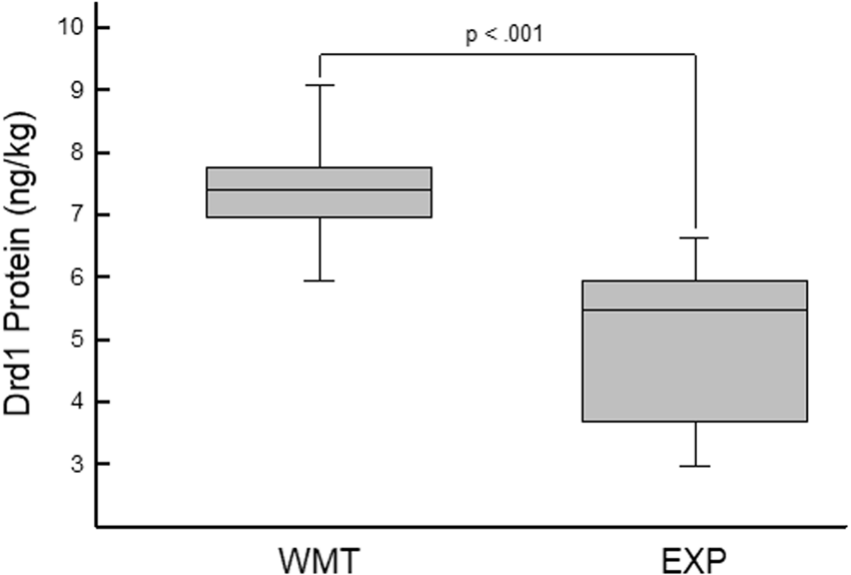
Utilization of working memory increases D1 protein levels. Expression level of D1 receptors in the prelimbic cortex of animals that were either exposed to an intense working memory task (WMT; described in Fig. 4) or simple exposure (EXP) to the training apparatus with no explicit cognitive demands. The brackets indicate the range of values in each group, the median is marked by the horizontal line within the box, and the box spans the interquartile range of data points. An independent samples t-test revealed a significant difference in the expression level of D1 receptors with animals that were imposed to the working memory training regimen having an enhanced expression level of D1 receptors in the prelimbic cortex (p < 0.001). This result suggests that D1 receptors can be made rapidly available to accommodate cognitive demands.

Two hours after the completion of the second session of working memory training, animals were sacrificed, brains were rapidly extracted and a gross extraction of the mPFC was performed. Analysis of the expression level of D1 receptors revealed a significantly higher density of D1 protein in animals that had recently engaged in working memory implementation relative to those that were in a state of cognitive rest, t(26) = 5.617, p < 0.001 (See Fig. 5). As suggested by the results of Experiment 1, this result indicates that the D1 receptor can rapidly be made available to accommodate cognitive demands.

## Discussion

The present set of experiments provides evidence of a positive relationship between the rate of turnover/recovery of newly synthesized D1 dopamine receptors in the mPFC and animal’s general cognitive abilities (GCA). For this analysis, we circumvented the receptor recycling pathway by utilizing the alkylating agent EEDQ, an irreversible dopamine receptor antagonist. By utilizing an irreversible antagonist, each receptor that was bound by the ligand ultimately undergoes degradation instead of re-sensitization and re-insertion into to the plasma membrane. Thus receptors present after 24, 72, and 288 hours after the EEDQ injection were receptors inserted into the membrane after the administration of the antagonist (with the exception of receptors that were not bound by the ligand).

At any given time, there exists in the ER a balance between incompletely-folded proteins that are retained in the ER and fully-folded proteins that are trafficked out of the ER for insertion into the plasma membrane. Such restricted trafficking provides the cells with a pool of functional receptors that can be called upon immediately by relaxing the scrutiny of the quality control system or stabilizing receptor conformation^24^, and previous work has indicated that large reserves of dopamine D1 and D2 receptors are present in the PFC, particularly in young animals^28^. In addition to rate of turnover, here we assessed whether an increase in D1 receptor turnover rates in high GCA animals could in part be modulated by the ER membrane-associated protein DRiP78. Levels of DRiP78 have been shown to regulate the dynamic interplay between the number of membrane bound receptors and a cell’s receptivity to transmitter binding. DRiP78 regulates the number of membrane bound receptors through binding to the receptors carboxyl terminal hydrophobic motif FxxxFxxxF in the ER. The FxxxFxxxF motif of the D1 receptor has been shown to function as an ER export signal. Levels of DRiP78 are known to be proportional to the number of D1 receptors at the plasma membrane in an inverted U shaped curve fashion, and an optimal level of DRiP78 is needed for efficient D1 trafficking^25,32^.

Higher expression of DRiP78 is associated with an increase in the retention of the D1 receptors in the ER^32^, which can lead to a larger pool of intracellular receptors that could potentially be available to undergo maturation during a time of high cognitive demand. The present results show that during a time of cognitive rest, high GCA animals express an enhanced expression level of DRiP78. Thus it can be postulated that high GCA animals have an intracellular pool of immature receptors at their disposal for times of intense cognitive demands. During times of high cognitive loads, DRiP78 could release its tonic hold on the immature receptors in the ER thus allowing them to undergo maturation and be inserted into the membrane. With the availability of an intracellular pool of immature receptors, the delay between full maturation and plasma membrane binding would be shorter than undergoing full synthesis of novel proteins, thus rapidly accommodating cognitive demands.

In humans and mice, innate differences in general cognitive ability as well as differences consequent to working memory training are associated with increased sensitivity of dopamine D1 receptors in areas of the PFC^16,18^. In the present analysis, we determined that this increase in sensitivity is associated with an increase in the density of D1 receptors. Newly synthesized receptors are generally more sensitive to ligand binding^33^, and receptor use is known to facilitate the rate of recycling^34,35^. Thus not only is an increased rate of turnover likely to account for the observed increased sensitivity of the D1 receptor that is associated with higher general cognitive performance, working memory training (which stimulates these receptors) is likely to similarly increase their rate of turnover and/or density. As rate of turnover is influenced by use and critically modulates receptor sensitivity, this modulation of rate may serve as the point of convergence between genetic regulation of GCA and the impact of cognitive experience (e.g., working memory training) on general cognitive performance.

Since a receptor’s sensitivity is in part determined by the time since its synthesis (for review, see ref.^24^), an increase in the rate of turnover of the D1 receptor may be a mechanism by which its sensitivity is modulated. Moreover, while the pool of reserve D1 receptors is known to be high in young animals^28^, the rate of D1 receptor turnover is markedly reduced in aged brains^30^, and thus rate of turnover may be the common link between receptor sensitivity, innate variations in intelligence, and cognitive aging. This possibility is particularly exciting in light of evidence that rate of receptor turnover is use-dependent, and can thus account for the effects on D1 receptor sensitivity of working memory training (e.g.^36^), which can be effective as a means to mitigate cognitive aging^37,38^.

Prior reports in humans using positron emission topography (PET), magnetic resonance imaging (MRI), and post mortem studies have demonstrated that an age-related decline in dopamine D1 binding potential and/or sensitivity exists between young and aged cohorts^39–42^. Coinciding with this loss in D1 binding potential is the impairment in executive functioning which has been identified as a primary modulator in deficits of working memory, cognitive flexibility, and overall general cognitive performance. This phenomenon has been similarly characterized in animals^43–45^, suggesting that across species, aging is associated with a global decrease in the binding potential of D1 receptors. It would be intriguing in light of the current evidence, taken collectively with past studies, to begin examining the effects that prolonged cognitive training regimens would have on the signaling capacity of D1 receptors and its effects on age-related cognitive declines.

## Materials and Methods

### EXPERIMENT 1

#### Subjects

Ninety-eight CD-1 outbred male mice were obtained from Harlan Laboratories (Indianapolis, IN). This strain exhibits a high degree of behavioral and genetic variability (comparable to wild populations), and thus are well suited for experiments examining individual differences. The mice arrived in our laboratory between 70–80 days of age at which time their weights ranged from 25–30 grams. The mice were singly housed in clear standard shoe box cages in a temperature and humidity controlled vivarium which was maintained on a 12 hour light/dark cycle. In order to minimize any differential stress responses exhibited by the animals due to experimenter handling, the animals were removed from their cages and held by an experimenter for 90 sec/day, five days per week, for a period of two weeks prior to the start of behavioral testing.

#### Learning Battery

To quantify animals’ general cognitive abilities, we evaluated animals’ performance on a battery of four diverse tasks that impinge on different domains of learning, sensory/motor, and motivational systems. All of the animals were tested on the tasks in the following order: Lashley III Maze, water maze, odor-guided discrimination, and passive avoidance. Three days of rest intervened between each successive task in the learning battery. For tasks utilizing food reinforcers, animals were food deprived 48 hours prior to training by allowing only 90 min of access to food per day (delivered within two hours of the end of the light cycle).

Lashley III Maze: The Lashley III maze was constructed of a start box, four connected alleys, and a goal box that could contain a food pellet. (For an illustration of the Maze, see ref.^5^. Efficient performance in the maze requires that the animal make five spatial alternations to reach the goal box. Over trials, the number of errors (i.e., wrong turns or retracing) committed by an animal typically decrease. The maze was constructed of black Plexiglas. Each alley measured 58 × 6 cm, and has 16 cm high walls. A goal box was (20 cm long) located 10 cm from the end of the last alley. A 2 cm diameter cup was located near the rear of the goal box, and 45 mg BioServe pellet (rodent grain) served as a reinforcer. The floor of the maze was illuminated at 80 Lux. The maze was isolated behind white Plexiglas to minimize any extra-maze landmark cues.

Animals were food-deprived and then acclimated and trained on two successive days. To familiarize animals with the reinforcer, each animal received three BioServe food pellets in its home cage on the day prior to acclimation. On the acclimation day, each mouse was placed for 4 min in each alley of the maze, but the openings between the alleys were blocked (with black Plexiglas) so that the animals could not through the maze. Three reinforcers were present in the food cup during this acclimation. On the subsequent training day, each animal was placed in the start box and allowed to navigate the maze until it reached the goal box and consumed the single food reinforcer that was present. After consuming the reinforcer, the animal was returned to its home cage for a 20 min intertrial interval (ITI). The entire apparatus was cleaned with a mild alcohol solution during the ITI, after which, the animal was returned to the start box to begin the next trial. This sequence was repeated for five trials. On each trial, errors (a turn in an incorrect direction, including those which result in path retracing) to enter the goal box were recorded.

Odor Guided Discrimination: Mice easily learn to use odors to guide their search for food. Here, mice were placed in a square field in which food cups were located in three corners and were marked by distinct (e.g., almond, lemon, mint). Food was present in each cup, but was only accessible to the mice in the cup marked by the odor of mint. Each mouse was placed in the empty corner of the field, and allowed to retrieve the food pellet in the cup marked by the mint odor. On each trial, the food cups were located in a new location (corner),, but the accessible food was always marked by mint. On successive trials, mice made fewer search errors directed at the cups in which food was not available, and near errorless performance is typically observed within 2–4 training trials.

A 60 cm square field was constructed of black Plexiglas and had 30 cm high walls. The field was located in a dimly lit (10 fc) room with a high rate of air turnover (3 min volume exchange). Three 4 × 4 × 2.0 cm (l, w, h) aluminum food cups were located in three corners of the field. The food reinforcer (30 mg of chocolate-flavored puffed rice) was placed in a 1.4 cm deep, 1 cm diameter depression in the center of each cup. The food in two of the cups was covered (1.0 cm below the surface of the cup) with a wire mesh so that it was not be accessible to the animal, while in the third cup (marked by the mint odor), the food could be retrieved and consumed. A 3 cm long cotton-tipped laboratory swab was located between the center and rear corner of each cup.

Prior to each trial, 25 ul of either lemon, almond, or mint odorants (McCormick flavor extracts) were added to fresh cotton swabs on each food. The mint odor was always present on the cup that held accessible food. On the training day, each mouse received four training trials with three food cups present. On each trial, the mouse was placed in the empty corner of the field, and the trial continued until the animal retrieved and consumed the food from the cup marked by the mint odor, after which the mouse was left in the chamber for an additional 20 sec. The mouse was then returned to its home cage where a 6 min ITI ensued. Trials 2–4 proceeded in the same manner, but the locations of the food cups were rearranged, although the accessible food was always marked by the mint odor. On each trial, an error was recorded if an animal made contact with, or when its nose crossed a plane parallel to the perimeter of an incorrect cup.

Spatial Water Maze: In this task, mice are placed in a round pool of water from which they can escape onto a hidden (i.e., submerged) platform. Across trials, the animal’s path to the escape platform becomes more efficient. Starting each trial from a new location mitigates egocentric navigation and promotes dependence on spatial landmarks. Thus performance in this maze is said to reflect the animals’ representation of their environment as a “cognitive map”.

With our procedures, mice exhibit significant reductions in their latency to locate the escape platform within 3–6 training trials. To promote this rapid acquisition, animals were confined for 6 min in a clear Plexiglas cylinder on the submerged platform on the day prior to training, a longer ITI (10 min) was used than is typically (c.f., 90 sec), and the maze, surround, and water were black and visual cues were comprised of patterns of lights.

A round pool (140 cm diameter, 56 cm deep) was painted black on all surfaces and was filled to within 24 cm of the top with water made opaque with nontoxic, water soluble, black paint. An 11 cm diameter black platform was hidden in a fixed location 1.5 cm below the water’s surface midway between the perimeter and center of the pool. A ceiling-high black curtain surrounded the pool. Five different shapes (landmark cues) were variously positioned on the interior of the curtain at heights (relative to water surface) ranging from 24–150 cm. One of the cues was constructed of two adjacent 7 W light bulbs. The remaining shapes were comprised of strings of white LEDs (at 2.5 cm intervals). These shapes formed an “X” (66 cm arms crossing at angles 40° from the pool surface), a vertical “spiral” (80 cm long, 7 cm diameter, 11 cm revolutions), a vertical line (31 cm long) and a horizontal line (31 cm long). A video camera was located on the ceiling above the center of the pool (180 cm above the surface of the water). These visual cues provided the illumination of the maze (172 Lux at the water surface).

One day prior to training, each animal was placed on the escape platform and confined there for 360 sec. Training proceeded on the following two days. Six training trials were administered on Day 1 of training. On each trial, the animals were placed in the pool at one of three unique start locations. One starting point was located along the outer wall of the pool in the middle of each of the three quadrants other than the one that contained the submerged escape platform. The starting location alternated between the three available quadrants on each trial. An animal was judged to have escaped from the water when all four of its paws were on the platform, provided that the animal remained on the platform for at least 5 sec. Ten min intervened between each trial, during which time the animals were held in a warming cage. A 90 second limit on swimming was imposed on each trial. Any animal that had not located the escape platform within 90 sec was placed by the experimenter onto the platform, and then removed from the pool 10 sec later.

Animals were observed on a remote video monitor, and their performance was recorded for subsequent analysis. Day 2 of training proceeded as Day 1, although only four trials were administered. Each animal’s path length to the platform served as the index of that animal’s performance.

One-Trial Passive Avoidance: Animals will learn to avoid contact with aversive stimuli. “Passive avoidance” response is exemplified in step-down avoidance procedures, whereupon stepping off of a platform the animals encounters a foot shock, resulting in an increase in the animal’s latency to leave the safe platform. In the present procedure, the mouse was exposed to a compound of bright light and loud oscillating noise (rather than shock) when it stepped off the platform. This variant of this task has been found to support learning after only a single trial.

A dimly lit (20 Lux) chamber was used for training and testing. A circular (“safe”) (10 cm diameter, 8 cm high) had walls and floor constructed of white Plexiglas (the floor being comprised of plastic rods (2 mm diameter) arranged to form a pattern of 1 cm square grids). The ceiling of this chamber was translucent orange Plexiglas. A clear exit door (3 cm square) could slide horizontally to open or close the safe chamber. The bottom of the exit door was 4 cm above the floor of another circular chamber (20 cm diameter, 12 cm high). This “unsafe” chamber was constructed of clear Plexiglas and had a floor constructed of 4 mm wide aluminum planks arranged to form 1.5 cm grids oriented at a 45° angle relative to the grid floor in the safe chamber. When an animal stepped from the safe chamber onto the floor of the unsafe chamber, a compound aversive stimulus was initiated. This stimulus was comprised of a bright white light (550 Lux) and “siren” (Radio Shack sound oscillator, model 273–057; 60 dB above the 50 dB background, 2.4–3.7 kHz).

Each mouse was placed on the safe platform behind the closed exit door. After 4 min of confinement, the door opened and the latency of the animal to step onto the floor of the lower compartment was recorded. Prior to encountering the aversive stimulus, step-down latencies typically range from 6–12 sec. Upon contacting the floor of the lower chamber, the aversive stimulus (light + siren) was presented for 3 sec. Upon encountering the aversive stimulus, mice reliably retreat into the upper safe chamber, at which time the platform door was closed. The mice were then confined to the safe chamber for another 4 min, after which the door was opened and the latency to contact the lower grid floor was again recorded. The ratio of post-training to pre-training step-down latencies was calculated for each animal and served as an index of learning. We have previously found that performance after a single training trial reflects (on average) sub-asymptotic learning.

#### Analysis of General Cognitive Abilities of Individual Mice

As described in previous reports^5,46^, each animal received a performance score for each learning task, and that score represented its rate of acquisition on that task. To describe each animal’s general cognitive performance, the performance on each learning task was entered into a Pearson’s product-moment correlation matrix. Positive correlations across all comparisons would indicate that a common source of variance influenced the performance of animals across all tasks. The Pearson’s correlation matrix was then subjected to a principal components analysis, which provides an estimate of the amount of variance in aggregate performance that can be accounted for by a general factor. Based on the primary (general) factor from this analysis, a factor score was derived for each individual animal. A factor score is analogous to an average z-score for each animal’s performance on each learning task, where the z-score for each task is weighted according to its loading on the primary factor. These factor scores provide an estimate of each animal’s general cognitive ability relative to the other animals in the sample.

#### Assessing D1 Receptor Turnover Rate

Two weeks following the completion of the learning battery, three distinct (non-adjacent) groups were formed based on their general cognitive performance (i.e., factor scores). To form these distinct groups, groups were formed from the top 29, middle 23, and bottom 28 animals from the 98 animals that contributed factor sores. The nine animals between the middle and high categories and the 9 animals between the low and middle categories did not contribute to any further analyses. (These group sizes were chosen based on the number of samples that could be loaded on an ELISA plate.) The top and bottom third of animals would be administered EEDQ, while the middle third would receive no drug and would serve to establish a normal baseline from which to compare changes in D1 protein and Drip78 levels. The high (n = 29) and low (n = 28) GCA animals were given an intraperitoneal (i.p.) injection of *N*-ethoxylcarbonrl-2-ethoxy-1,2-dihydroquinoline (EEDQ; an irreversible full dopamine antagonist) at a dose of 8 mg/kg, and the animals from the middle of the distribution (n = 23) were given an i.p. injection of the vehicle. A dose of 8 mg/kg was chosen because in preliminary experiments it showed high receptor blockade (~70% @ 6 hours post injection) with only a low mortality rate (<6%). Control animals (from the middle of the GCA distribution) received comparable injections of saline. To determine the rate at which new receptors replaced those that had been irreversibly blocked, the animals were sacrificed by rapid decapitation at three different time points after EEDQ injection: 24, 72, and 288 hours. Either 9 or 10 animals from each group were sacrificed at each time point. (High GCA 24 hours group had 10 animals; High GCA 72 hours group had 10 animals; High GCA 288 hours group had 9 animals; Low GCA 24 hours group had 9 animals; Low GCA 72 hours group had 10 animals; Low GCA 288 hours group had 9 animals.) The brains were rapidly extracted and a gross extraction of the medial prefrontal cortical (mPFC) area was performed. Once the mPFC was extracted it was placed into an ice-cold lysis buffer (10 mM Tris-HCL pH 7.4, 1% (v/v) NP-40, 150 mM NaCl, 5 mM EDTA, 50 mM NaF, 1 mM phenylmethylsulfonyl fluoride; PMSF), containing a protease inhibitor cocktail (Cat# 78430, Thermo Scientific). Brain extracts were then purified by sonication, centrifuged at 15,000 rpm for a period of 10 minutes at 4 °C. The supernatants were then extracted, flash frozen in liquid nitrogen, and stored at −80 °C until further use.

In order to quantify the number of D1 receptors in each sample, an enzyme-linked immunosorbent assay was performed on a pre-coated anti-drd1 plate (Cat# MBS269095, MyBioSource). This antibody for the D1 receptor binds to the receptor’s C-terminus. Since this binding site is typically occupied by DRiP78 in sub-membrane immature receptors, those receptors awaiting transport to the membrane are predominantly undetected. Briefly, the standards and samples were loaded in duplicates onto the plate and allowed to incubate at 37 °C for 90 minutes. Once the incubation time period elapsed the plate was washed three times using the washing solution provided by the manufacturer. After the completion of the washes a biotinylated Mouse DRD1 antibody was added to each well and allowed to incubate at 37 °C for 60 min. The plate was then washed again three times, after which an enzyme conjugate was loaded into each well and once again allowed to incubate at 37 °C for a period of 30 min. Once this time period elapsed the plate was washed five times and the color reagent supplied by the manufacturer was added. The absorbance at 450 nm was measured with a microplate reader (Multiskan MCC, Thermo Fisher) in order to determine the level of D1 receptors present in each sample.

#### Assessing DRiP78 Expression Levels

DRiP78 expression levels were assessed on the same samples that contributed to the analysis of receptor turnover. In order to quantify the expression level of DRiP78 in each sample; an enzyme linked immunosorbent assay was performed on a pre-coated anti-DRiP78 plate (Cat# MBS924030, MyBioSource). Briefly, the standards and samples were loaded onto the plate in duplicates and allowed to incubate for two hours at 37 °C. Following the incubation, the standards and samples were removed from the plate and the Biotin-antibody was loaded into each well (with no intervening washes) which was then allowed to incubate for 60 min at 37 °C. Following the 60 min incubation, a series of five washes using the washing buffer provided by the manufacturer was performed. HRP-aviden was then loaded into each well and allowed to incubate for one hour at 37 °C. The plate was then washed and the TMB substrate was added to each well and incubated for 15–30 minutes at 37 °C. The stop solution was then added and the absorbance at 450 nm was measured using a microplate reader (Multiskan MCC, Thermo Fisher) in order to determine the levels of DRiP78 in each sample.

The reported values of D1 protein and DRiP78 are normalized relative to animals that did not receive EEDQ injections, i.e., that received vehicle injections, but that had previously been tested in the battery of learning tests. Thus reported values of 100% can be considered the baseline.

### Experiment 2

#### Subjects

32 genetically heterogeneous CD-1 male mice were obtained from Harlan Laboratories (Indianapolis, IN) at 65–80 days and a weight range of 24–29 grams. Housing and maintenance were as described in Experiment 1.

#### Quantifying General Cognitive Abilities

To characterize their general cognitive abilities, all animals underwent testing in the four-task learning battery described in Experiment 1. The procedures for implementing the learning battery were identical to Experiment 1 with the exception of the number of training trials. In this experiment all animals were trained to an asymptotic level of performance in order to ensure that any differences in the expression level of D1 receptors was a consequence of the imposition of a working memory training regimen, rather than as a consequence of different levels of learning. Animal housing and maintenance conditions were identical to that of the first experiment.

#### Working Memory Taxation

After completion of testing on the learning battery, animals were segregated into two groups that were matched for their GCAs (i.e., they were comprised of animals of similar mean and range of general cognitive abilities; see results). One group then received working memory training (working memory training; WMT, n = 16), and the second group received an equivalent amount of time in the training apparatus without explicit working memory training (exposure; EXP, n = 16). The procedures and apparatus are described below, and the rationale for this this task is described in detail elsewhere^4,10,11,47^. In short, the animals are required to alternate choices between two distinct radial arm mazes that share a common set of extra-maze guidance cues. This requires that the animals maintain a memory of prior choices under conditions of interference imposed by working in two mazes simultaneously.

Two 8-arm radial mazes were constructed of either black or grey Plexiglas. They both consisted of an 18 cm diameter central hub surrounded by eight 40 × 5 cm arms with a 0.75 cm lip around the perimeter of each arm. Each maze was raised 26 cm above the floor. At the end of each arm was a 1 cm depression in the arm which could be used as a food cup. Each food cup had a 1 mm hole at the bottom which led to a cup filled with 84 mg (6 pieces) of inaccessible food. This inaccessible food provided a scent of food and mitigated animals’ use of the scent of food to find cups in which food was accessible.

In addition to their color, the two mazes were further distinguishable. The black maze had an 18 cm tall clear Plexiglas enclosure surrounding its central hub, with each arm accessible through a 5 cm wide and 4 cm tall entrance door. The gray maze included no such enclosure around the central hub. The two mazes were placed side-by-side in the same testing room and thus shared a set of overlapping extra-maze visual cues (a pattern of LED lights as well as architectural features) which the animals could use to guide their navigation within the mazes.

At the start of a typical training trial in either the black or grey maze, the animal was placed in the central hub of the maze and allowed to navigate freely until it successfully found and consumed the Bio-Serv 14 mg dustless pellet located in the food cup at the end of each arm. Mice were judged as choosing an arm when their hind paws crossed the initial edge of each arm. Errors were scored when an animal chose an arm from which food had already been collected.

Training/testing in these mazes occurred over approximately seven weeks and was conducted in six phases, with two days off between each successive phase. In Group WMT, the final phase of testing was intended to make strong demands on the animals’ use of working memory. During all training/testing, animals were food deprived by providing them with only 90 min/day access to food in their home cages at the end of the light cycle (and thus were approximately 16 hours deprived at the start of any training/test session).

Initial training was performed on both groups of animals. All of the animals were trained first in the black maze. Initially, animals were acclimated to the maze for two consecutive days (**Phase 1**). On the first day of acclimation, animals were placed on each of the eight arms of the maze for 90 s without access to the central hub (entry doors were closed). One piece of food was available in each of food cups at the end of each arm. On the second day of acclimation, the animals were placed in the central hub of the black maze, and allowed access to each arm individually (by opening a door to one arm at a time). After emergence into the arm, animals were confined to the arm for 10 min, and were again allowed to collect a food pellet located in the cup at the end of the arm.

In **Phase 2**, animals were trained in the black maze, with one trial per day, for five days. Animals started in the central hub with all doors open, and allowed to navigate the maze until all eight food pellets were collected. Errors were recorded throughout the trial.

In **Phase 3**, animals were trained in the (gray) maze, one trial per day, for five days. Training proceeded in the same manner as in the black maze, but the acclimation phase was omitted (since animals were already acclimated to the mechanics of maze navigation).

In **Phase 4**, training continued for four days as in the prior two phases, but animals alternated between the black and gray maze on successive days. They were trained in the black maze on the first and third of four days, and on the grey maze on the second and fourth of four days.

In **Phase 5**, animals performed in both mazes on the same day. They were trained in the black maze early during the light cycle (3–4 hrs after lights on), then after a 4 hr ITI, were trained again in the grey maze. This training proceeded for four days.

All of the above training sessions were intended to have the animals trained to asymptotic performance on both mazes, and to have them performing two trials per day (one trial in each maze). At the end of these five phases of training, the animals were prepared for the critical phase (**Phase 6**) in which working memory would be utilized by Group WMT. In previous reports, we have observed that the working memory task implemented here is initially difficult for mice, although they can improve considerably over 12 days of training^31^. In the present experiment, our goal was not to train improvements in working memory, but rather, to determine the effect of intense utilization of working memory on D1 receptor protein density. In our prior work, we have observed that mice performed poorly on the working memory task through the initial two days of training (i.e., working memory was severely taxed). Thus in the present study, animals participated in this Phase 6 working memory task (or the control procedure) for just two days.

During the two days of working memory implementation, animals in Group WMT (working memory training) received one test trial per day, during which they alternated their choices in the black and grey mazes. They began each trial in the black maze, where they were allowed to make three correct choices (plus any errors). They were then be moved by the experimenter into the grey maze where they were allowed to make their first three correct choices (plus any errors). They were then returned to the black maze to make their next three correct choices (plus any errors), then again to the grey maze to make their next three correct choices (plus any errors). This alternation was then repeated for the final two correct choices in the black and grey mazes. Thus by the end of a trial, the subjects had obtained all 16 food reinforcers from both mazes (8 in each maze). Again, this procedure is thought to heavily tax working memory capacity, since the animals must maintain a memory of choices in one maze while subjected to the interference associated with choices in the second maze (where both mazes share common extra-maze search cues).

On the two days in which Group WMT performed in the working memory task, Group EXP (exposure) was simply placed in the central hub of each maze for the same amount of time and with eight food pellets placed in the center of the hub. Under these conditions, the animals could experience the mazes and collect food with no requirement for the implementation of working memory.

#### Assessing D1 Receptor Expression Levels

Two hours after the final working memory testing session (when animals will have been in a state of high cognitive demands), their brains were rapidly extracted and a gross extraction of the mPFC was performed. Quantification of the expression level of D1 receptors was identical to the quantification of D1 receptor levels described in Experiment 1, however, in this case, basal protein levels were to be determined, so the animals were not treated with a D1 receptor antagonist prior to their sacrifice.

### Data availability

The datasets generated during and/or analyzed during the current study are available from the corresponding author on reasonable request.

### Use of experimental animals

This work was approved by the Rutgers University IAUCUC committee (Protocol # 98–024) and all experiments were performed in accordance with relevant guidelines and regulations.
